# Neighbourhood perceptions of physical activity: a qualitative study

**DOI:** 10.1186/1471-2458-8-101

**Published:** 2008-03-28

**Authors:** Louise N Burgoyne, Catherine Woods, Rosarie Coleman, Ivan J Perry

**Affiliations:** 1 Department of Epidemiology and Public Health, Brookfield Health Sciences Complex, University College Cork, Ireland; 2 School of Health and Human Performance, Dublin City University, Ireland; 3 Northside Community Health Initiative, Harbour View Road, Knocknaheeny, Cork, Ireland

## Abstract

**Background:**

Effective promotion of physical activity in low income communities is essential given the high prevalence of inactivity in this sector.

**Methods:**

This study explored determinants of engaging in physical activity in two Irish city based neighbourhoods using a series of six focus groups and twenty five interviews with adult residents. Data were analysed using constant comparison methods with a grounded theory approach.

**Results:**

Study findings centred on the concept of 'community contentment'. Physical activity was related to the degree of contentment/comfort within the 'self' and how the 'self' interacts within the neighbourhood. Contemporary focus on outer bodily appearance and pressure to comply with societal expectations influenced participants' sense of confidence and competence. Social interaction, involvement, and provision of adequate social supports were viewed as positive and motivating. However normative expectations appeared to affect participants' ability to engage in physical activity, which may reflect the 'close knit' culture of the study neighbourhoods. Access to suitable local facilities and amenities such as structured and pleasant walking routes was regarded as essential. Indeed participants considered walking to be their preferred form of physical activity which may relate to the minimal skill requirement, ease of access and low financial costs incurred.

**Conclusion:**

In the context of physical activity, health promoters need to be conscious of the difficulties that individuals feel in relation to bodily appearance and the pressure to comply with societal standards. This may be particularly relevant in low income settings where insufficient allocation of resources and social supports means that individuals have less opportunity to attend to physical activity than individuals living in higher income settings.

## Background

Physical inactivity is a major public health concern. Vulnerable sectors associated with lowest levels of leisure time activity include those with low levels of education and income [1-3]. There are limited theoretically based qualitative studies exploring participation in physical activity and sport [4]

Descriptive research with adult populations has shown fitness, relaxation, skills development, challenge and enjoyment among the determinants of physical activity [5]. Psychological theories facilitating explanation of the adoption and maintenance of physical activity include self-efficacy theory [6-10] and the theory of planned behaviour [11-15]. Of late, research has focused on the influences of social-ecological environments [16-18].

Promotion of physical activity is challenging. Public health products comprising behavioural and social changes are often placed in markets with negative demand [19,20]. Additionally, changes to physical activity recommendations and subsequent changes to corresponding health messages over the past three decades, have resulted in some public confusion [21]. Further confusion is evident in societies pre-occupation with outer appearance where contemporary standards dictate 'a lean, toned exercising body' [22]. Health promoters face a considerable task in conveying the message that current recommendations advocating a minimum of thirty minutes of moderate intensity accumulated physical activity on all or most days of the week are straightforward to achieve.

Effective health promotion necessitates an understanding of target populations. We carried out an exploratory study on determinants of physical activity in two adjacent low income neighbourhoods in Cork city, Ireland. There were three objectives: to understand perceptions of physical activity, to determine how residents felt about neighbourhood physical activity and to provide information for development of a tailored physical activity intervention [23].

## Methods

Physical activity intervention design and implementation can benefit from meaningful participation of the community and relevant agencies [24-26]. Our study engaged the partnership of a local health agency (Northside Community Health Initiative) serving the neighbourhoods of Knocknaheeny-Hollyhill in Cork. This collaborative approach facilitated the process of purposive sampling and participant recruitment, through the agencies extensive local knowledge and respected presence in the community. The study comprised of six focus groups and twenty-five unstructured interviews accompanied by a standard topic guide.

Interview and focus group questions were determined by the project researcher, community health workers, and an anthropologist working at the Department of Epidemiology and Public Health, UCC at the time of the study. Focus groups and interviews were moderated by the researcher (L.B.) and a community health worker (R.C.). Each participant was provided with a voucher for £20 in recognition of their contribution to the study.

### Participants

Purposive sampling was used to recruit adult participants (Table 1). Sampling was facilitated through the health agency staff utilising their extensive local knowledge. Consideration was given to inclusion of information rich cases that would reflect the population diversity. Factors included: sex, approximate age, levels of physical activity, marital status, dependents and interaction with the local health agency. Levels of physical activity were based on the American College of Sports Medicine guidelines for health. Potential participants were sampled from lists of residents held at the health agency. Others were identified using names provided by associates such as the clergy and child care workers.

**Table 1:** Characteristics of Focus Group and Interview Participants

	Participants (N)	RA*	NRA**	NICHE***	< 40 yo
Group 1 (men)	7	3	4	4	4
Group 2 (women)	5	2	3	2	3
Group 3 (women)	16	7	9	8	9
Group 4 (men)	6	3	3	3	2
Group 5 (women)	12	6	6	7	6
Group 6 (men)	9	4	5	5	4
Interviews (Men)	11	5	7	6	5
Interviews (Women)	14	8	6	7	7

* Regularly Active ** Not regularly active ***Have had involvement with local health agency prior to this study.

Potential recruits were contacted by letter and phone call. When they were not in a position to participate, letters were forwarded to other candidates. Those who could not attend stated that this was for time, family or work reasons. Participants gave informed consent for the research. The research was considered and approved by the Cork Teaching Hospitals Research Ethics Committee

### Materials

Topics were determined by the project researcher and community health workers. These included: residents preferred forms of physical activity; perceived personal benefits; factors which encourage and discourage physical activity and views on local facilities availability and their usefulness to the neighbourhoods. Focus groups and interviews were recorded using audio tape and comprehensive note taking.

### Focus Group Procedures

Focus groups were hosted at the health agency premises in May and June 2001. Each session took place in a room used by residents for community events/meetings, and was approximately one hour's duration. After initial introductions, topics were posed to the group. Participants were encouraged to give their points of view and to clarify responses. When there was a lull or a pause in conversation, probes were used to stimulate discussion. Those who were shy or less inclined to respond were encouraged.

### Unstructured Interview Procedures

Unstructured interviews were completed between September and November 2001. A majority of these were held in residents' homes (N = 20) with the remainder hosted at the health agency (N = 5). Interviews were between thirty and sixty minutes duration depending on the interviewee's availability. As for the focus groups, participants were encouraged to give their views centring on the topics provided by the interviewer. Probes were used where necessary.

### Analytic Tools

Analysis was carried out using constant comparison methods with a grounded theory approach. The Anno Tape 1.0 solution together with paper systems were used to manage and code the findings. Open, axial and selective coding techniques were employed [27]. Concepts, themes and their properties and dimensions, were identified. Initially each recording was listened to in terms of its general content. Open coding was used to 'fracture' the data or break it down into meaningful phrases, sentences or words. These were subsequently grouped into sub-categories and categories. Axial coding facilitated the process of reassembling the fractured data and was used to uncover relationships between categories and subcategories. Selective coding was used to integrate the data to reach a 'central category', explaining the main theme of the research.

Since similar categories developed from both the focus groups and the interviews, results are reported together. Combined reporting of focus group and interview data can be useful when collecting the same kind of data from the same kinds of participants [28,29]. Data coding was cross checked by an independent researcher.

## Results

### Central and Major Themes

Data analysis uncovered three main themes: competence and confidence, social interaction and involvement, and facilities and the physical environment. The central theme of this study was 'community contentment'. Being physically active was related to the degree of contentment/comfort within the individual and how the individual interacts with the social and physical environment. Findings are illustrated with quotations followed by pseudonyms and grouping in parentheses (Tables 2, 3, 4).

**Table 2:** Theme of 'competence and confidence' and supporting verbatim examples

Theme	Verbatim Examples
Competence and Confidence	"It's good for your mind, your body too. Sometimes I get stressed, I do go out of my mind here with stress and I just go off playing a match, its great then, exercise is very good for you" (Joan, interview 9).
	"If you are gone forty years of age and you start running on concrete, you'll do damage, you know, the bones are going to get brittle. Fast walking is the best thing or hill-walking or something like that." (John, focus group 4)
	"I hate, you know, in a gym now, I wouldn't be interested in that, because when you go you have to know what you are doing. I just don't like the gym anyway" (Sara – interview 6).
	"I think you know though, in a gym now, I think you really have to know what you are doing. It's intimidating, you would be conscious of yourself" (Mary – interview 3)

**Table 3:** Theme of 'social interaction and involvement' and supporting verbatim examples

Theme	Verbatim Examples
Social Interaction & Involvement	"I'd love to be able to play, you know play tennis because I think it's a sort of a social game as well as exercise, ..........I think, but another one would be volleyball, its another sort of a team game which is social enough as well" (James, focus group 5)
	"I'm in (name of gym) six or seven years now and I'll tell you it's the greatest thing for any fellow I'll tell you that, you know the fellows, you know the girls, you'll have a laugh and exercise when you want, it's the greatest thing ever" (Jack, focus group 4).
	"If there was a community based building where there was expertise inside in the building that could actually get people in a group situation and turn around and say look, this is what we will do Monday, this is what we will do Wednesday and this is what we will do Friday and draw out a plan for people that combines exercise with a healthy lifestyle, that is what is needed" (Eddie, focus group 4)
	"I'm living here and I don't know of anything going on around the place. You might get the paper the odd time and you might see something but there is nobody there to tell you, whether it's that I am in the wrong place at the wrong time I don't know" (Kate – focus group 6)

**Table 4:** Theme of 'facilities and the physical environment' and supporting verbatim examples

Theme	Verbatim Examples
Facilities & Physical Environment	"It's beautiful there, it's outstanding like, but not the way it is, you are walking over cars and trucks and vandals and everything. If there was a system in place it would be as good a walk as any.....if you could get around the dogs that are running up there!" (Suzanne, focus group 6)
	"They go for a few weeks and then they drop off and then they are moaning that there is nothing going on. Now that really gets me because there are great things going on" (Sally, interview 14).
	"The club facilities aren't suitable......If fifteen players walk in to train and there are people in there, the players are going to take over". (Joe, focus group 2)
	"We need something like a big facility. I mean there are fields, there are fields all over the place. It would be grand if we did get something now, something for the kids, and the mothers could get some time on their own that way, or a big crèche and people might get out then" (Marie, interview 25)

### Competence and Confidence

Regular physical activity resulted in benefits on mood and ability to deal with day to day situations constructively. Residents spoke about feeling good after engaging in activity, and how this can act as an "antidote" to negative moods. Stress or tension relief was given as a primary benefit. Increases in energy levels were also cited.

Residents who were not regularly active felt that they were "too unfit" to begin activity. Effort expenditure would be higher than for an active person: "I am drained if I go for a walk!" Linked to this were thoughts about aging and the tendency to decrease physical activity with age. These feelings were equated with a lack of energy, fear of falling or fear of going out alone. Walking was cited as being an effective form of activity for older adults.

Issues on confidence in gym or exercise classes emerged. Approximately half of the residents (regularly and not regularly active) said they felt uncomfortable and tended to avoid such environments. Concerns were centred on performance and operation of equipment in front of peers. For women this was particularly during exercise classes (aerobics, circuits etc). Men felt that there was a degree of "competition between men" at gyms and pressure to perform.

Connections were made between feeling confident in a gym/class and acquired levels of skill and knowledge. Several residents commented that they could not attend a swimming pool because they had never learned to swim properly. Others expressed a lack of interest in classes (aerobics, circuits etc.) because they were unsure about the sequences involved. Men felt they could not attend exercise classes since these are "designed for women".

Women were concerned about body shape and size. Several equated physical activity directly with body weight. They also expressed a need for fashionable attire and training shoes, and some stated that they could not afford these. They felt that in order to do physical activity you have to: "look the part, wear the right clothes". Indeed thoughts of "being looked at" were strongly related to body image. Both men and women were conscious of being observed during an activity, whether in the gym, the pool or in a class. Feelings of self-consciousness were related to views regarding the close knit nature of the neighbourhood where "everyone knows everyone else". Stemming from this several residents said they prefer to do activities outside the locality.

Regularly active residents spoke about physical activity becoming a routine, an integral part of their day. Men were dominant with regard to the compulsion of activity and with feeling compelled to maintain routines: "they call me the road runner; I can only relax when I get back". In this instance, missing out on activity caused guilt or irritability. Some likened physical activity to a drug or religion. For others it was linked to personal health in that they became active after a period of ill health or a scare. Activity thus became important for health maintenance.

Residents' preferred physical activity was walking followed by swimming and attending a gym. Walking was considered versatile, easy to perform and suitable for all age groups: "walking is just straightforward like". It was also seen as a way of benefiting from being outdoors, enabling people to "get out into the fresh air" and was regarded as a "natural and healthy type of activity".

### Social Interaction and Involvement

Most residents said they enjoyed company when doing an activity and liked to plan activities with others. Walking with neighbours, going to exercise classes with friends, or joining a club with a friend was viewed as motivating. Social aspects of attending gyms or leisure facilities were very positive for some.

Women stated that they often walk in groups with friends: "we go about four nights, sometimes five nights a week". However, whilst most were positive about group activities, some residents preferred lone activities because they did not want to depend on others: "if you want something done, you got to do it yourself".

Views on sociable activities led to views about preceding or following activities with social gatherings e.g., going to an exercise class and meeting for coffee afterwards. Several residents remarked that although they would like to "unwind" socially after a class or activity, existing facilities are inadequate. A general opinion was that gym and leisure centre environments need more areas for members to meet and relax.

The topic of clique's highlighted negative aspects of group based activity. Some men felt they could not use certain gyms/clubs in neighbouring communities because they were not regular attendees. For women, cliques were associated with class environments: "they are all in their own little groups". A few spoke of their reluctance to attend classes on account of other participants. Several found group situations difficult in general. Having to perform physical activity within a group exacerbated their difficulty. These residents felt they were not good at interacting with others and associated group based activity with people who are.

Access to expert support and advice on physical activity was seen as critical. Suggested advisers included gym instructors, class instructors and doctors or teachers. Residents expressed that gym/leisure centre instructors are difficult to approach or else they do not appear to care: "they will put you on the bike and that's the last you will see of them". They felt that a community-based facility would provide a much-needed neighbourhood resource in terms of support, education and advice.

The need for supports from significant others emerged. This need was more frequently expressed by women who said that support regarding child minding and housework was an enabling factor for physical activity. They also felt that a positive attitude towards activity on the part of spouses, children and significant others is supportive. Linked to the topic of supports were the financial costs associated with facilities memberships and exercise classes for those on minimum incomes. Walking was noted as an activity with little associated cost. However it was stressed that improving amenities for walking in the area would require investment.

Awareness of locally based opportunities for physical activity was low. Many residents felt that there was an overall lack of neighbourhood information. In contrast, a minority said that there is information if people look for it, and highlighted advertising and resources provided by the local health agency. These residents felt that other locals do not become sufficiently involved in neighbourhood activities choosing to use outside facilities instead. Either this or they cease attendance at local activities after a few sessions.

### Facilities and the Physical Environment

A majority of residents thought additional facilities for physical activity were needed: "there's nothing, no swimming, no gyms", "we only have a road". Existing facilities were viewed as limited and in need of renovation. The lack of facilities meant using a car or buses to attend gyms/classes: "you have to take two buses to get to the gym". Transport was a problem for residents from one-car families or those who primarily use public transport. All residents felt that existing areas for walking in the neighbourhood were in need of repair else new routes were required. Attention was drawn to a particular walking route that has been used for many years and is currently neglected.

A minority view with respect to available facilities was that activities were organised but not accessed: "no-one uses anything that is set-up". Residents who held this view felt that locals choose to complain instead. This view was connected to a minority opinion suggesting local football facilities (gym, pitch etc.) could be usefully accessed by locals. However most felt that these cannot cater for general activity needs. Other residents drew attention to increasing insurance costs as a barrier to accessing such facilities, particularly in low-income areas.

Neighbourhood cleanliness was linked with doing local activities. It was generally felt that the physical environment was unclean and this was de-motivating: "the glass and broken bottles around the place are just unnatural". Lack of cleanliness was not an issue for some however, who were aware of it but said it would not stop them from walking recreationally. There were strong views on domestic refuse. Some residents had experienced rubbish being dumped near their homes: "They are dumping rubbish over my daughters' wall! There was a sense of disillusionment with regard to refuse, with remarks about burned out cars, empty bottles and cans (from people drinking outside) and discarded appliances such as fridges. With respect to walking, residents also spoke about specific local areas that were not safe. These included areas with poor lighting and places frequented by gangs of youths.

## Discussion

Findings revealed that greater degrees of contentedness within the individual, and with the local social and physical environment indicate a higher likelihood of being physically active.

Several factors relating residents' competence and confidence with physical activity were identified. Positive factors included mental wellbeing, mood regulation and increased energy levels. Negative factors included a perceived lack of ability on the part of not regularly active residents and more generally, feelings of intimidation associated with gyms or exercise classes. Concerns about body weight and attire were dominant among women whilst men were conscious of competitive atmospheres. Positive and negative factors were evident where physical activity was incorporated into daily schedules, but similarly induced feelings of guilt or compulsion. Encouraging features of social interaction and involvement included enjoyment, group based activity, friendship, family and professional supports. De-motivating aspects included pressure to perform and clique environments. Most residents noted a lack of local information pertaining to activities, whilst a minority felt that there was sufficient information if sought. These views were related to the general view that there are insufficient facilities, and the contrasting minority view that activities are organised but are not accessed by the population. Environmental cleanliness was highly relevant for outdoor activity. Walking was the activity of choice for most residents which may reflect a lack of facilities for other forms of physical activity, and may also be influenced by the minimal competency required.

Positive findings relating to residents' competence and confidence are consistent with documented psychological benefits of physical activity on health [5,30] and quality of life [31-33]. Residents' perceived lack of ability and self consciousness relates to self-efficacy [6], an effective behavioural determinant [8,9,34]. Residents' feelings may also reflect public confusion about health messages regarding levels of activity needed to maintain health [21]. In addition, they may reflect societal standards placing emphasis on outer appearance, dictating a fit body as the ideal [35,36]. Rather than comply with unattainable social expectations for physical activity performance, people may choose to abstain.

Motivation to comply with perceived expectations of others is defined as subjective norm within the theory of planned behaviour [11,12]. This model is a useful predictor of physical activity [13,37,38]. In the current study, subjective norm appeared to influence residents' ability to engage in activity which may relate to the cohesive nature of the neighbourhoods. Although the literature reports smaller effect sizes between subjective norms and intentions than for other constructs [39-41], subjective norms may be more influential in neighbourhoods with low migration. The possible influence of subjective norms on physical activity engagement in such neighbourhoods is worthy of further investigation.

There is an urgency to address physical inactivity within socially excluded sectors [42]. In this study, residents who took regular activity discussed how it becomes routine, and some equated feelings of guilt with missing scheduled sessions. Integration of physical activity with daily life is a public health goal incorporating the need for personal discipline. However, health promoters need to be mindful of balance in this context given the current tendency towards 'healthism'. Societies' emphasis on appearance and pressure to conform to contemporary ideals has been translated into activities such as dieting, exercise and 'weight watching' [22].

Enjoyment was an important motivating aspect of physical activity which is supported by the literature [4,43-45]. Another motivator was social support. Residents indicated that this can be obtained from significant others. Friendship and family support has been shown to influence physical activity [46-48]. Other supports discussed included those obtained from professionals. Indeed physical activity counselling support has been shown to be effective [49,50]. Having supportive others to talk, ask questions or receive honest feedback is critical to initiating and maintaining behaviour change [51]. In this study, lack of supports meant residents felt less in control of their ability to do regular activity. Given the lower income status of the study neighbourhoods, social supports and resulting sense of personal control are of particular importance. People who are under resourced do not have the same opportunities for leisure and other 'risk' factors as those with higher levels of resources [52].

Group based activities were motivating for most residents. Positive characteristics include a communication network, shared goals and rules of behaviour culminating in a sense of identity or loyalty [53]. However, group activity was not viewed positively by a minority who expressed feelings of intimidation when having to perform structured routines. Group 'motivational climates' may influence physical activity [5]. For example, a 'performance' climate is one where class participants are compared, anxiety is felt about making mistakes and praise is given for superior performance. Such a climate may be exacerbated between peers from 'close knit' localities such as the study neighbourhoods. Indeed residents viewed cliques as unconstructive since they increased feelings of intimidation. Present day consumer culture which fosters unrealistic standards for physical appearance may also be influential again here.

Most residents noted a lack of information about neighbourhood activities and expressed a need for publicity. Some felt that there was sufficient information on activities if it was sought. However these were also aware of events organised by the local health agency, and at the time of the study advertising was primarily done by the health workers. Indeed community health projects are noted for their work in providing services that are not facilitated by the statutory authorities [54].

Access has been reported to influence physical activity [17,55,56]. In this study the majority view was that there were insufficient neighbourhood facilities and amenities. Parents stressed the need for additional child minding services, linking to the aforementioned issue of required social supports. In opposition to this, a few residents felt strongly that there were indeed facilities in place and that people were not using them. Discussion by the authors with the health workers confirmed that there were few facilities available to the local population.

Walking was the favoured physical activity for a majority of residents. Indeed it is reported as the preferred form of physical activity in the EU [2] and in Ireland [57]. Preferences for walking were related to enjoyment of outdoor activities and minimal financial costs. They may also be related to a desire for a form of physical activity outside the disciplined gym/class. With respect to walking areas, residents stressed the need for well-maintained routes, and how existing routes required improvements to make them more 'walkable'. This is supported by the literature indicating that maintenance of footpaths and infrastructure is associated with higher levels of walking [16,58,59]. There was considerable mention by residents of cracked pavements, broken glass, burned out cars and dumping of domestic waste which contributes to making the locality an unpleasant and dangerous place to pursue activity. Environmental safety is shown to be associated with walking behaviour [55,56,60]. Aesthetics and attractiveness of amenities are associated with physical activity [57,17].

There are several study limitations. Since it involved residents from two adjacent neighbourhoods in one city, results cannot be generalized. Neither can selection bias be ruled out. Those who agreed to take part in the groups did so because they had the time and the inclination. Researcher bias cannot be ruled out since both the authors have professional interests in health promotion and community development. Despite efforts to remain objective and to allow the participants' to direct discussions towards issues of personal relevance, this may have impacted upon the results. Categories were cross-checked for coding and interpreted by an independent researcher to assess their relevance with the data. It may also have been useful to use respondent validation since respondents reactions to emerging findings can help to refine explanations [61].

## Conclusion

Findings from this study centre on the concept of 'community contentment'. If individuals are content personally, socially and within their physical environment, then they are more open to being physically active. Psycho-social influences on physical activity included self-efficacy, social pressure and expectations and social support. Physical environmental determinants included access to facilities and amenities together with availability of clean areas for walking and recreation.

With regard to promotion of physical activity, health professionals face a challenge in generating balance in the midst of a consumer culture focused on bodily appearance. When considering the determinants of physical activity it is important to remain conscious of the pressures faced by individuals in this context. More work is required to develop theoretical frameworks informing health promotion interventions, programmes and campaigns [4]

As mentioned in the Introduction one of the reasons for this exploratory study was to obtain guidance in design of a physical activity intervention tailored to the study neighbourhoods. Findings thus influenced the decision to introduce a neighbourhood walking initiative [23], the Irish Heart Foundations' international 'Path to Health', and to include self-efficacy [6], personal control, subjective norm [11,12] and environmental safety [62] amongst the study measures.

## Competing interests

The author(s) declare that they have no competing interests.

## Authors' contributions

LB participated in the design, data collection, analysis, and drafting of the manuscript. RC was involved in the design, coordination and data collection. IJP conceived of the study and helped to draft the manuscript. CW participated in the analysis and drafting of the manuscript. LB, IJP and CW read and approved the final manuscript. RC was on sick leave at this time.

## Pre-publication history

The pre-publication history for this paper can be accessed here:


